# The outcomes of surgical ASD closure in the era of catheter ASD closure; experience of single institute

**DOI:** 10.1186/1749-8090-8-S1-P141

**Published:** 2013-09-11

**Authors:** S Kubota, T Hoashi, K Kagisaki, S Yazaki, J Kobayashi, I Shiraishi, H Ichikawa

**Affiliations:** 1 Departments of Cardiovascular surgery, National Cerebral and Cardiovascular Center, Suita, Japan; 2 Department of Pediatric Cardiac surgery, National Cerebral and Cardiovascular Center, Suita, Japan; 3 Department of Pediatric Cardiology, National Cerebral and Cardiovascular Center, Suita, Japan

## Background

There is still demand for surgical closure of secundum atrial septal defect (sASD) in spite of the wide-spread of Amplatzer Septal Occluder (ASO). We sought to investigate the outcome of surgical sASD closure after ASO was introduced in our institute to find the indication and its morbidity compared with ASO.

## Methods

Between August 2005 and December 2012, 524 patients with ASD below 18 years old were enrolled in this study. ASO was performed in 328 and surgical closure was performed in 195. The indication for surgery was insufficiency of BW (<15kg) in 61, morphological reason in 120, attempted but failed ASO in 8 (including one emergency case) and AV valve regurgitation in 5.

## Results

There was no mortality. Only one patient needed re-exploration for acute cardiac tamponade and four patients needed permanent pacemaker implantation. Eleven required blood transfusion (BTF) (5.6%). Cerebral infarction was encountered in one patient.

The patient was divided into two groups according to the body weight less than (group A) or above (group B) 15 kg.

In group A, surgery was performed through partial sternotomy with small incision (5.2 ± 1.2 cm). The mean size of ASD was 20.6 ± 8.4 mm. Nine patients who weigh less than 9.0 kg required BFT. No other patients required BTF.

In group B, The mean size of the ASD was 22.6 ± 8.4 mm. Since 2009, partial sternotomy with limited skin incision (6.3 ± 1.5 cm) was applied in most of the patients (55 out of 66). Two patients required BTF for decreased hematocrit during cardiopulmonary bypass.

## Conclusion

Although the surgical closure of ASD is safe and mostly done without transfusion, post-operative cerebral infarction occurred in one out of 192 patients. The physicians must inform the advantage and disadvantage of both interventions carefully to patients and families before deciding the treatment option.

